# Flares after hydroxychloroquine reduction or discontinuation: results from the Systemic Lupus International Collaborating Clinics (SLICC) inception cohort

**DOI:** 10.1136/annrheumdis-2021-221295

**Published:** 2021-12-15

**Authors:** Celline C Almeida-Brasil, John G Hanly, Murray Urowitz, Ann Elaine Clarke, Guillermo Ruiz-Irastorza, Caroline Gordon, Rosalind Ramsey-Goldman, Michelle Petri, Ellen M Ginzler, D J Wallace, Sang-Cheol Bae, Juanita Romero-Diaz, Mary Anne Dooley, Christine Peschken, David Isenberg, Anisur Rahman, Susan Manzi, Søren Jacobsen, Sam Lim, Ronald F van Vollenhoven, Ola Nived, Andreas Jönsen, Diane L Kamen, Cynthia Aranow, Jorge Sanchez-Guerrero, Dafna D Gladman, Paul R Fortin, Graciela S Alarcón, Joan T Merrill, Kenneth Kalunian, Manuel Ramos-Casals, Kristján Steinsson, Asad Zoma, Anca Askanase, Munther A Khamashta, Ian N Bruce, Murat Inanc, Michal Abrahamowicz, Sasha Bernatsky

**Affiliations:** 1 Division of Clinical Epidemiology, Research Institute of the McGill University Health Centre, Montreal, Quebec, Canada; 2 Division of Rheumatology, Department of Medicine and Department of Pathology, Queen Elizabeth II Health Sciences Centre, Halifax, Nova Scotia, Canada; 3 Center for Prognosis Studies in the Rheumatic Diseases, Toronto Western Hospital, University of Toronto, Toronto, Ontario, Canada; 4 Divisions of Clinical Immunology/Allergy and Clinical Epidemiology, University of Calgary, Calgary, Alberta, Canada; 5 Autoimmune Diseases Unit, Hospital Universitario Cruces, Barakaldo, País Vasco, Spain; 6 Rheumatology Research Group, University of Birmingham, Birmingham, UK; 7 Medicine/Rheumatology, Northwestern University, Evanston, Illinois, USA; 8 Rheumatology, Johns Hopkins University, Baltimore, Maryland, USA; 9 Medicine/Rheumatology, SUNY Downstate Medical Center, New York City, New York, USA; 10 Cedars-Sinai/David Geffen School of Medicine at UCLA, Cedars-Sinai Medical Center, Los Angeles, California, USA; 11 Rheumatology, Hanyang University, Seongdong-gu, Seoul, The Republic of Korea; 12 Immunology and Rheumatology, Instituto Nacional de Ciencias Médicas y Nutrición Salvador Zubiran, Ciudad de Mexico, Mexico; 13 Medicine, Unversity of North Carolina, Chapel Hill, North Carolina, USA; 14 Division of Rheumatology, University of Manitoba, Winnipeg, Manitoba, Canada; 15 Division of Medicine, University College London, London, UK; 16 Rheumatology, University College London, London, UK; 17 Allegheny Singer Research Institute, Allegheny Health Network, Pittsburgh, Pennsylvania, USA; 18 Copenhagen Lupus and Vasculitis Clinic, Center for Rheumatology and Spine Diseases, Rigshospitalet, Copenhagen, Denmark; 19 School of Medicine, Emory University, Atlanta, Georgia, USA; 20 Department of Rheumatology, Amsterdam Rheumatology and Immunology Center, Amsterdam, The Netherlands; 21 Rheumatology, Lund University, Lund, Sweden; 22 Faculty of Medicine, Department of Clinical Sciences Lund, Section of Rheumatology, Lund University, Lund, Sweden; 23 Medical University of South Carolina, Charleston, South Carolina, USA; 24 Northwell Health Feinstein Institutes for Medical Research Institute of Health Innovations and Outcomes Research, Manhasset, New York, USA; 25 Center for Prognosis Studies in the Rheumatic Diseases, Toronto Western Hospital, Toronto, Ontario, Canada; 26 University of Toronto, Toronto, Ontario, Canada; 27 Medicine—Rheumatology, Université Laval, Quebec, Quebec, Canada; 28 Department of Medicine, Division of Clinical Immunology and Rheumatology, University of Alabama at Birmingham Center for Health Promotion, Birmingham, Alabama, USA; 29 Arthritis and Clinical Immunology Program, Oklahoma Medical Research Foundation, Oklahoma City, Oklahoma, USA; 30 Division of Rheumatology, Allergy and Immunology, University of California San Diego School of Medicine, La Jolla, California, USA; 31 Department of Autoimmune Diseases, Universitat de Barcelona, Barcelona, Catalunya, Spain; 32 Rheumatology, Department of Obstetrics and Gynecology, Landspitali University Hospital, Reyjavik, Iceland; 33 Lanarkshire Centre for Rheumatology, Hairmyres Hospital, East Kilbride, South Lanarkshire, UK; 34 Rheumatology, Columbia University Medical Center, New York, New York, USA; 35 The Rayne Institute, St Thomas Hospital, St Thomas' Hospital, London, UK; 36 Arc Epidemiology Unit, The University of Manchester, Manchester, UK; 37 Department of Internal Medicine, Division of Rheumatology, Istanbul University, Fatih, Istanbul, Turkey; 38 Department of Epidemiology Biostatistics and Occupational Health, McGill University, Montreal, Quebec, Canada; 39 Division of Rheumatology, McGill University Health Centre, Montreal, Quebec, Canada

**Keywords:** systemic lupus erythematosus, hydroxychloroquine, autoimmune diseases, epidemiology

## Abstract

**Objectives:**

To evaluate systemic lupus erythematosus (SLE) flares following hydroxychloroquine (HCQ) reduction or discontinuation versus HCQ maintenance.

**Methods:**

We analysed prospective data from the Systemic Lupus International Collaborating Clinics (SLICC) cohort, enrolled from 33 sites within 15 months of SLE diagnosis and followed annually (1999–2019). We evaluated person-time contributed while on the initial HCQ dose (‘maintenance’), comparing this with person-time contributed after a first dose reduction, and after a first HCQ discontinuation. We estimated time to first flare, defined as either subsequent need for therapy augmentation, increase of ≥4 points in the SLE Disease Activity Index-2000, or hospitalisation for SLE. We estimated adjusted HRs (aHRs) with 95% CIs associated with reducing/discontinuing HCQ (vs maintenance). We also conducted separate multivariable hazard regressions in each HCQ subcohort to identify factors associated with flare.

**Results:**

We studied 1460 (90% female) patients initiating HCQ. aHRs for first SLE flare were 1.20 (95% CI 1.04 to 1.38) and 1.56 (95% CI 1.31 to 1.86) for the HCQ reduction and discontinuation groups, respectively, versus HCQ maintenance. Patients with low educational level were at particular risk of flaring after HCQ discontinuation (aHR 1.43, 95% CI 1.09 to 1.87). Prednisone use at time-zero was associated with over 1.5-fold increase in flare risk in all HCQ subcohorts.

**Conclusions:**

SLE flare risk was higher after HCQ taper/discontinuation versus HCQ maintenance. Decisions to maintain, reduce or stop HCQ may affect specific subgroups differently, including those on prednisone and/or with low education. Further study of special groups (eg, seniors) may be helpful.

**Key messages**

**What is already known about this subject?**

- In clinical practice, patients often ask physicians about hydroxychloroquine (HCQ) reduction or discontinuation.
- The literature and clinical experience suggest that HCQ reduction/withdrawal may be safe in some stable patients, but in other settings it may be associated with disease flare.

**What does this study add?**

- Using real-world data from an international systemic lupus erythematosus (SLE) inception cohort, maintaining HCQ was associated with a lower flare risk than when reducing or stopping HCQ, even in patients with low disease activity or remission.
- Low education was associated with increased flare risk among patients discontinuing HCQ.
- Patients with SLE on prednisone or immunosuppressors were at higher risk for flare.
- The crude flare rate was over 30 flares per 100 person-years, even while maintaining HCQ.
- Over the interval of follow-up, most patients experienced a flare.
- This emphasises the ongoing need to optimise therapeutic options in SLE.

**Key messages**

**How might this impact on clinical practice or future developments?**

- If a patient were to ask “if someone decreases HCQ, what are the chances of flaring sooner than if they stay on the same dose?” the physician could reply: “According to this research, there is a 54% probability that a given person decreasing HCQ will flare sooner than someone staying on the same dose.”
- Similarly, our results suggest that overall, a given patient who stops HCQ has a 61% probability of flaring sooner than a given patient who continues on HCQ. (Ssee Spruance et al (PMC478551) on how to interpret hazard ratioHRs in terms of chances).
- This translates back to the crude flare rates: maintaining HCQ had about 30–31 events per 100 person- years, while those that reduced or stopped HCQ had about 40–41 events per 100 person-years.
- Decisions to maintain, reduce or stop HCQ may affect specific subgroups differently, including those on prednisone and/or with low education.

## Introduction

Hydroxychloroquine (HCQ) is a cornerstone of systemic lupus erythematosus (SLE) treatment.^1–3^ However, physicians and patients often consider reducing or discontinuing HCQ over the decades-long course of SLE, sometimes in order to limit cumulative exposure and avoid important HCQ-induced toxicity.^4 5^

Over 20 years ago, a pivotal HCQ withdrawal randomised trial suggested that sustained HCQ might greatly reduce disease flares, leading to the suggestion that all patients should remain on HCQ ‘indefinitely’.^6 7^ However, it is hard to know if results from that trial apply to patients in whom physicians would want to taper treatment, notably those in remission or very low activity, since many of the patients in the trial did not have completely controlled disease at study entry (40% were using prednisone and the average SLE Disease Activity Index score was 8).^7^ For years, physicians have attempted to identify a subgroup of patients in whom it would be safe to stop or reduce HCQ, such as seniors.^8^

The aims of our study were to determine (1) the extent to which HCQ reduction or discontinuation is associated with increased risk of SLE flares, and (2) the predictors of a flare once HCQ is reduced or discontinued, using a longitudinal international SLE inception cohort.

## Methods

### Data source

The Systemic Lupus International Collaborating Clinics (SLICC) cohort is a multinational inception cohort for SLE outcomes research.^9^ From 1999 to 2011, a cohort of recently diagnosed patients with SLE was recruited from 33 SLICC sites in Europe, Asia and North America.^10^ Briefly, patients meeting American College of Rheumatology revised classification criteria for SLE^11^ were enrolled within 15 months of diagnosis. Data are collected per protocol at enrolment and annually and entered into a centralised database.

### Study population and design

We selected all patients on HCQ therapy at baseline (cohort entry) or during the follow-up up to April 2019. At each annual follow-up visit, average HCQ daily dose since the last assessment was recorded. We evaluated outcomes in patients reducing/stopping HCQ and compared them with those remaining on therapy. Patients contributed person-time in the HCQ maintenance cohort until they either reduced the dose, discontinued treatment, had the outcome of interest or were censored (death, lost to follow-up or end of study, April 2019), whichever came first. If HCQ was reduced, patients contributed person-time in the HCQ reduction cohort until they either discontinued HCQ, had the outcome or were censored. If HCQ was discontinued, patients contributed person-time in the HCQ discontinuation cohort until they had the outcome or were censored. A given patient could contribute person-time to one or more cohorts.

**Figure 1:**
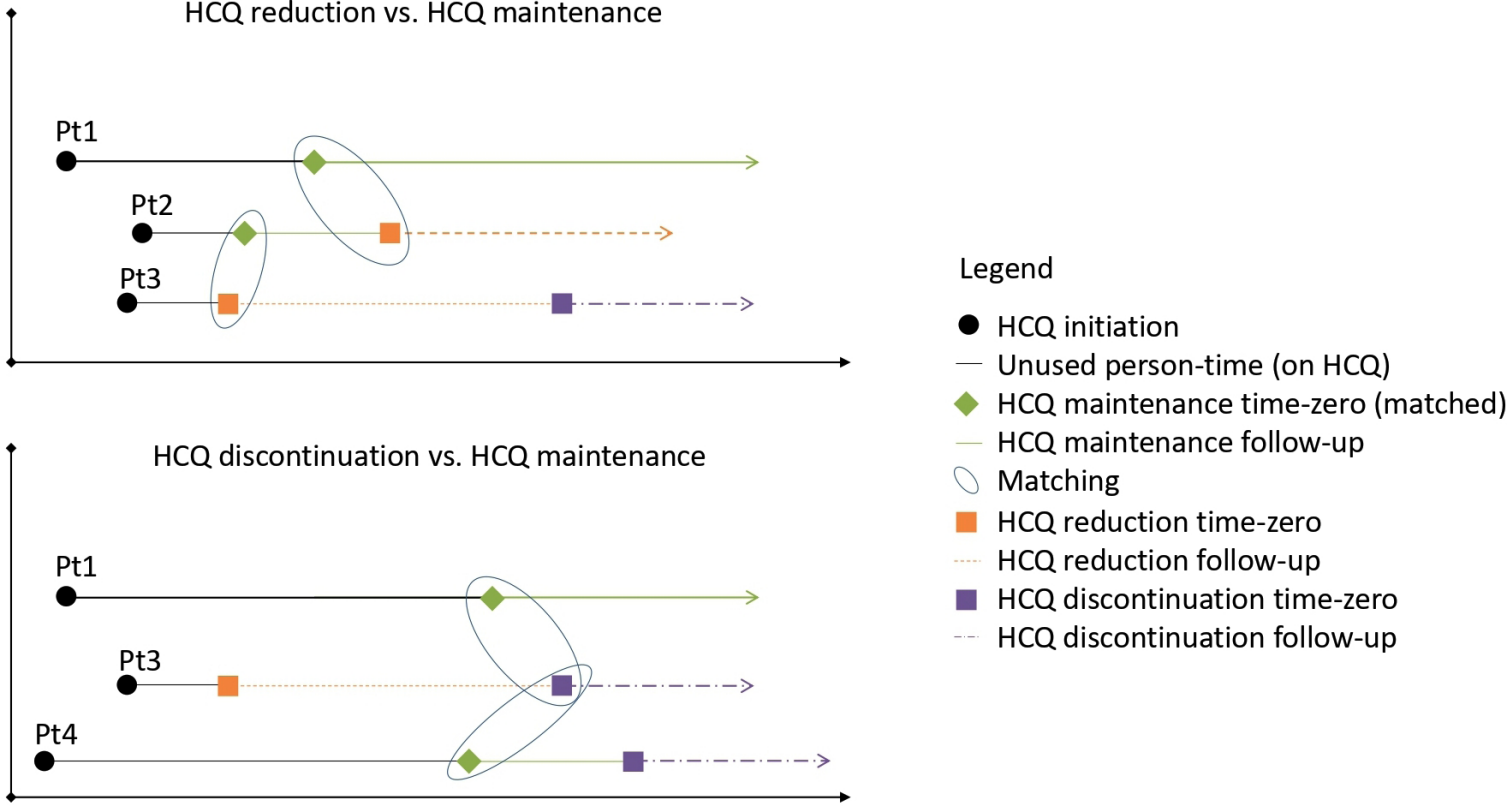
Example of four cohort patients (Pt1–4). A given patient could contribute person-time to one or more cohorts. Hydroxychloroquine (HCQ) maintenance person-time was matched (2:1) to the reduction or discontinuation cohorts on HCQ duration at time-zero.

Time-zero among those reducing HCQ was the first date recording HCQ reduction and time-zero in the HCQ discontinuation cohort was the first date recording discontinuation. To create the comparison HCQ maintenance groups (one for reduction, one for discontinuation), each patient reducing or discontinuing HCQ was randomly matched on prior HCQ use duration to up to two individuals remaining on HCQ treatment (figure 1). A time-zero was then assigned to the matched maintenance group on the day of matching. This approach balances the groups on the length of previous treatment at the beginning of follow‐up and avoids immortal person-time.^12^

Patients who discontinued HCQ but started chloroquine immediately were not included in the discontinuation cohort, as they were still on an antimalarial; these were censored at the time of switching.

The reasons underlying HCQ dose change were not recorded, but dose reduction may have been due to the following scenarios: (a) physician or patient may have been concerned about cumulative use of HCQ and/or lowered dosing to reflect guidelines (particularly the 2016 American Academy of Ophthalmology (AAO) guidelines, which cautions against dosage >5 mg/kg/day)^5^; (b) low SLE activity; (c) other reasons (eg, intolerance, patient request). Reasons for stopping HCQ may include (a) retinal toxicity; (b) clinical disease remission; (c) non-adherence; (d) intolerance, pigmentary skin changes or other adverse effect; (e) other reasons (eg, cost, healthcare access issues, drug insurance issues, patient choice).

We explored ways to categorise these possible reasons. Among patients who reduced HCQ dose, we identified how many had their dose reduced to 5 mg/kg/day after the 2016 AAO guidelines, and, of the remainder, how many had low disease activity state^13^ (SLE Disease Activity Index-2000 (SLEDAI-2K) <4 and current prednisone dose ≤7.5 mg/day). Patients not falling into one of these groups were classified into ‘other reasons’. Similarly, among those who stopped HCQ, we first identified those who had retinal damage on the SLICC Damage Index (SDI). Of those without retinal damage, we identified how many were in remission^14^ (SLEDAI-2K=0 and no prednisone or immunosuppressives in the last year). For the remainder, reasons were unclear but may reflect non-adherence or other unknown reasons.

### Outcome

The primary composite outcome was time to the first of the following events indicating a SLE flare: (a) increase of at least four points (above the score at time-zero) in the SLEDAI-2K^15^; (b) hospitalisation for SLE (eg, skin and joint flare, nephritis, pericarditis and pneumonia) and/or (c) augmented SLE therapy, defined as increased HCQ (or restart if discontinued) or a new start/increase in prednisone, immunosuppressive agents (azathioprine, methotrexate, mycophenolate mofetil, cyclophosphamide), biologics (rituximab or belimumab) or start of chloroquine. Quinacrine was used by only nine patients in our sample and was not considered as augmented SLE therapy. Since immunosuppressive agents may be given in addition to or instead of steroid therapy to lower the dose of steroids,^16^ we did not compute an event when patients increased/started an immunosuppressor (azathioprine, mycophenolate or methotrexate) but decreased their prednisone dose at the same visit. Hospitalisation data were available for 60% of patients and the composite outcome for patients without hospitalisation data was based on increase in disease activity and therapy augmentation only. Sensitivity analyses excluding hospitalisation from the composite outcome for all patients were also performed.

### Covariates

Decisions to reduce, stop or maintain HCQ may be driven by patient or clinical characteristics that are also associated with the outcome. Therefore, we considered potential confounders or effect modifiers, assessed at time-zero: sociodemographic variables (sex, Caucasian vs non-Caucasian race/ethnicity, high school education or less vs college/university education), age at SLE diagnosis (continuous) and geographic location (North America, Europe or Asia). Other variables assessed at time-zero included body mass index (BMI, continuous), current smoking (yes/no), high disease activity (≥4 points on SLEDAI-2K, a validated definition of active SLE),^15 17^ SLE duration (continuous, years), current prednisone (yes/no), current immunosuppressive agents (azathioprine, methotrexate or mycophenolate mofetil) and current biological agents (rituximab or belimumab), and presence of renal damage, based on the SDI.^18^

### Statistical analysis

In descriptive analysis at time-zero, we described the means and SDs for continuous variables and frequency distributions for categorical variables.

For the HCQ reduction/discontinuation cohorts and their respective maintenance control cohorts, we calculated crude incidence rates (first flare) with 95% CIs. A multivariable Cox proportional hazards (PH) model was used to estimate the adjusted HRs (HRs), with 95% CIs, for time to first flare in patients who reduced or discontinued HCQ (vs the maintenance groups), while controlling for the covariates listed above. Hazard proportionality was assessed using Schoenfeld and Martingale residuals.

Separate multivariable Cox PH models were estimated in the reduction, discontinuation and maintenance cohorts to assess which characteristics were associated with increased risk for first flare.

As a secondary analysis, we aimed to assess how disease activity status influence the risk of SLE flares after HCQ reduction or discontinuation (vs HCQ maintenance). Thus, we stratified the absolute flare rates and adjusted Cox models by low disease activity state or remission.

We also conducted sensitivity analysis. Since the same patient could contribute person-time to different cohorts being compared, we accounted for potential clustering by using random effects in our Cox models. Also, to evaluate the impact of having patients without complete outcome information (ie, missing hospitalisation data), we considered only increase in disease activity and therapy augmentation in the computation of the composite outcome for all patients.

All analyses were conducted with SAS V.9.4 (SAS Institute, Cary, North Carolina, USA).

### Patient and public involvement

Patients with SLE, patient advocates and organisations such as the Canadian Network of Improved Outcomes in SLE and the Canadian Rheumatology Association were engaged as partners since the early phases of our project, providing feedback on the protocol, interpretation of findings and dissemination. For instance, this study was planned and designed based on focus groups conducted in 2017 with patients with rheumatic disease,^19^ which identified that uncertainties about risks and benefits of stopping/continuing drugs were a primary concern. Our patient-partner assisted in the development of questionnaires and provided feedback regarding interpretation of findings. We also conducted interviews of individual patient with SLE to explore experiences and preferences with HCQ dose changes^20^ and defined potential reasons underlying HCQ changes, and incorporated this in our analyses, as mentioned before.

Lupus Canada and the Arthritis Society have pledged support to disseminate findings via websites, communiques and their provincial chapters. The Singer Family Fund for Lupus Research will help with knowledge dissemination through newsletters mailed twice yearly to patients with SLE.

## Results

Among the 1711 patients enrolled in the SLICC cohort, we included 1460 (85.3%) who initiated HCQ. We identified 592 patients who reduced HCQ (564 were matched to 778 patients maintaining HCQ) and 407 patients who discontinued HCQ (389 were matched to 577 patients remaining on HCQ). There were few differences in patient characteristics at time-zero between the matched groups: patients reducing HCQ were more likely to be from Asia and patients discontinuing HCQ were less likely to be Caucasian (table 1).

**Table 1:** Characteristics at time-zero of patients with SLE who maintained, reduced or discontinued HCQ

Characteristics at time-zero*	HCQ reduction n=564	HCQ maintenance n=778	HCQ discontinuation n=389	HCQ maintenance n=577
Female (%)	90.6	87.9	90.2	87.0
N missing	0	0	0	0
Race/Ethnicity (%)				
Caucasian	51.6	55.1	42.9	55.6
Asian	24.3	14.7	19.3	13.9
Black	12.4	16.1	15.4	15.9
Others	10.6	13.3	21.4	13.9
N missing	6 (1.1)	6 (0.8)	4 (1.0)	4 (0.7)
Age at SLE diagnosis (years, mean±SD)	34.1±13.4	35.6±13.3	33.6±13.4	35.9±13.6
N missing	0	0	0	0
No college/university education (%)	34.0	38.9	38.8	39.9
N missing	6 (1.1)	16 (2.0)	3 (0.8)	8 (1.4)
Geographic location (%)				
North America	56.2	63.2	59.6	62.6
Europe	26.1	27.9	26.5	29.3
Asia†	17.7	8.9	13.9	8.1
N missing	0	0	0	0
Time on HCQ (years, mean±SD)	3.4±2.6	3.2±2.5	4.2±3.2	3.9±3.1
N missing	0	0	0	0
HCQ daily dosage (mg, mean±SD)	240±73	347±83	0	349±81
N missing	0	0	0	0
SLE duration (years, mean±SD)	5.5±3.0	5.4±3.0	6.7±3.5	6.1±3.4
N missing	0	0	0	0
SLEDAI-2K ≥4 (%)	39.9	35.7	38.0	36.0
N missing	15 (2.6)	15 (1.9)	11 (2.8)	19 (3.3)
Renal damage (%)	6.4	5.7	10.7	5.4
N missing	3 (0.5)	5 (0.6)	5 (1.3)	2 (0.3)
Current smoker (%)	25.9	33.2	29.6	31.5
N missing	3 (0.5)	4 (0.5)	7 (1.8)	2 (0.3)
BMI (mean±SD)	24.1±5.1	25.6±5.9	25.1±5.7	25.7±5.9
N missing	16 (2.8)	30 (3.8)	7 (1.8)	23 (4.0)
Current prednisone (%)	58.0	55.4	51.9	52.8
N missing	0	0	0	0
Current immunosuppressors‡ (%)	44.1	47.0	41.6	46.8
N missing	0	0	0	0
Current biological agents§ (%)	3.0	2.6	3.6	4.0
N missing	0	0	0	0

*Time-zero of each subcohort (not inception cohort entry).

†Asia was represented by a single country, South Korea.

‡Immunosuppressors included mycophenolate, azathioprine and methotrexate.

§Biologics included belimumab and rituximab.

BMI, body mass index; HCQ, hydroxychloroquine; SLE, systemic lupus erythematosus; SLEDAI-2K, SLE Disease Activity Index-2000.;

The HCQ reduction or discontinuation was further classified according to the possible reasons for the respective changes. Specifically, we estimated that 5.0% may have reduced HCQ therapy as result of the AAO guidelines (daily dose changed from >5 mg/kg to 5 mg/kg after July 2016, based on real body weight), 54.8% because of low disease activity state and the remainder (40.2%) presumably reduced due to other reasons (eg, intolerance, patient preference, etc). Among those who discontinued HCQ, 4.4% had retinal damage, 15.2% were in remission and 80.5% may have stopped HCQ due to other reasons, including non-adherence and intolerance.

### SLE flares

The HCQ reduction cohort was followed for an average of 2.0 years per patient (with 78.7% flaring over follow-up, table 2) while the average follow-up in the other cohorts was about 1.7 years (with 72% flaring in the HCQ discontinuation cohort, and about 50% flaring in the maintenance cohorts). Need for therapy augmentation was frequent and hospitalisation due to lupus flares was relatively uncommon. The crude incidence rate of the first flare was considerably higher among those who reduced or stopped HCQ versus those who remained on the drug (table 2). Compared with HCQ maintenance, the adjusted HRs for SLE flare were 1.20 (95% CI 1.04 to 1.38) and 1.56 (95% CI 1.31 to 1.86) for the HCQ reduction and discontinuation cohorts, respectively. The mean doses of those reducing HCQ and flaring versus those reducing but not flaring were similar (data not shown).

**Table 2:** Incidence rates of the first flare in patients with SLE who maintained, reduced or discontinued HCQ

	HCQ reduction n=564	HCQ maintenance n=778	HCQ discontinuation n=389	HCQ maintenance n=577
First flare (any)				
Number of events (%)	444 (78.7)	413 (53.1)	280 (72.0)	292 (50.6)
Therapy augmentation only	399 (70.7)	325 (41.8)	252 (64.8)	239 (41.4)
Increase in disease activity only	61 (17.0)	127 (16.3)	68 (17.5)	81 (14.0)
Hospitalisation only	1 (0.2)	1 (0.2)	0	2 (0.5)
Total person-years in follow-up	1110.2	1294.7	677.9	973.4
Crude rate/100 person-years (95% CI)	40.0 (36.4 to 43.9)	31.9 (29.0 to 35.1)	41.3 (36.7 to 46.4)	30.0 (26.7 to 33.6)

HCQ, hydroxychloroquine; SLE, systemic lupus erythematosus.

### Risk factors

Separate multivariable Cox PH models were fit in each of the HCQ reduction, discontinuation and maintenance cohorts to estimate HRs for potential risk factors (table 3). Use of prednisone and immunosuppressives were both associated with higher risks of SLE flares in all cohorts (although in the discontinuation cohort, the 95% CI for the immunosuppressives HR just barely included the null value). We also observed a lower flare risk among patients reducing HCQ who live in Asia (South Korea) versus North American patients. Lower education was associated with an increased risk of SLE flares among patients who discontinued HCQ.

**Table 3:** HRs and 95% CIs for the first SLE flare, according to HCQ cohort

Characteristics at time-zero	HCQ reduction	HCQ maintenance	HCQ discontinuation	HCQ maintenance
	aHR (95% CI)	aHR (95% CI)	aHR (95% CI)	aHR (95% CI)
Male sex	0.93 (0.66 to 1.32)	0.96 (0.68 to 1.34)	0.97 (0.64 to 1.46)	0.77 (0.52 to 1.15)
Non-Caucasians	1.27 (1.00 to 1.61)	1.02 (0.81 to 1.28)	0.96 (0.70 to 1.32)	0.96 (0.73 to 1.27)
Age at SLE diagnosis in years	1.00 (0.99 to 1.01)	1.00 (0.99 to 1.01)	0.99 (0.98 to 1.00)	1.01 (1.00 to 1.02)
No college/university education	1.01 (0.82 to 1.24)	1.10 (0.90 to 1.36)	**1.43 (1.09 to 1.87**)	0.92 (0.72 to 1.18)
Geographic location				
North America	Reference	Reference	Reference	Reference
Europe	1.24 (0.98 to 1.59)	1.16 (0.91 to 1.48)	1.02 (0.75 to 1.37)	0.99 (0.75 to 1.31)
Asia	**0.70 (0.51 to 0.94**)	1.00 (0.69 to 1.43)	0.73 (0.49 to 1.08)	0.87 (0.56 to 1.34)
SLE duration	1.00 (0.96 to 1.04)	1.01 (0.98 to 1.06)	1.00 (0.96 to 1.04)	1.02 (0.98 to 1.06)
Active disease (SLEDAI-2K ≥4)	1.17 (0.95 to 1.44)	1.22 (0.98 to 1.51)	1.25 (0.95 to 1.64)	1.22 (0.95 to 1.56)
Renal damage	0.88 (0.57 to 1.37)	0.94 (0.58 to 1.53)	0.88 (0.60 to 1.30)	0.88 (0.49 to 1.56)
Body mass index	1.02 (1.00 to 1.05)	0.99 (0.98 to 1.01)	0.99 (0.97 to 1.01)	1.00 (0.97 to 1.02)
Smoker	1.07 (0.85 to 1.35)	0.88 (0.70 to 1.11)	1.02 (0.78 to 1.35)	0.94 (0.71 to 1.23)
On prednisone	**1.49 (1.16 to 1.91**)	**1.65 (1.28 to 2.13**)	**1.58 (1.15 to 2.17**)	**1.87 (1.38 to 2.54**)
On immunosuppressives	**1.37 (1.09 to 1.72**)	**1.84 (1.46 to 2.32**)	1.31 (0.96 to 1.77)	**1.84 (1.39 to 2.44**)
On biologics	0.72 (0.39 to 1.35)	1.00 (0.51 to 1.95)	0.70 (0.35 to 1.39)	0.77 (0.33 to 1.79)

Renal damage was defined as a score ≥1 in the SLICC/ACR Damage Index renal item (low glomerular filtration rate, proteinuria or end-stage renal failure). Prednisone, immunosuppressives and biologics were dichotomous variables (yes/no). Immunosuppressive drugs included azathioprine, mycophenolate and methotrexate. Biologics included belimumab, rituximab and abatacept.

Bolded values are those whose 95% CI excludes the null value.

ACR, American College of Rheumatology; aHR, adjusted HR; HCQ, hydroxychloroquine; SLE, systemic lupus erythematosus; SLEDAI-2K, SLE Disease Activity Index-2000; SLICC, Systemic Lupus International Collaborating Clinics.

### Secondary and sensitivity analyses

Table 4 presents the results from the prespecified secondary analysis restricted to subgroups of patients on disease activity status. We observed that maintaining HCQ was associated with lower SLE flare risk even for patients in low disease activity state or in remission at time-zero (table 4). Patients not in remission tended to have relatively higher crude flare rates, about 46–48 events per 100 patient-years when lowering or stopping HCQ, and about 39–41 events per 100 patient-years when maintaining HCQ.

**Table 4:** Adjusted HRs with 95% CIs for SLE flares associated with HCQ reduction/discontinuation versus maintenance: main and stratified analyses

	HCQ reduction versus maintenance			HCQ discontinuation versus maintenance		
	No. of patients	Absolute flare rate/100 person-years (95% CI)	Adjusted HR (95% CI)*	No. of patients	Absolute flare rate/100 person-years (95% CI)	Adjusted HR (95% CI)*
Main analysis	1342	40.0 (36.4 to 43.9) vs 31.9 (29.0 to 35.1)	1.20 (1.04 to 1.38)	966	41.3 (36.7 to 46.4) vs 30.0 (26.7 to 33.6)	1.56 (1.31 to 1.86)
Stratified analyses:						
Low disease activity‡ state at time-zero						
Yes	815	37.5 (33.2 to 42.4) vs 27.8 (24.5 to 31.6)	1.32 (1.10 to 1.60)	592	35.5 (30.4 to 41.3) vs 26.6 (22.8 to 30.9)	1.62 (1.28 to 2.05)
No	527	43.9 (38.0 to 50.6) vs 39.8 (34.3 to 46.1)	1.04 (0.84 to 1.29)	374	53.6 (44.7 to 64.2) vs 36.4 (30.5 to 43.5)	1.60 (1.22 to 2.09)
Remission† at time-zero						
Yes	196	26.2 (20.1 to 34.1) vs 13.2 (9.5 to 18.4)	2.14 (1.34 to 3.42)	133	24.7 (17.7 to 34.6) vs 12.2 (8.0 to 18.8)	2.77 (1.46 to 5.26)
No	1146	46.3 (41.9 to 51.1) vs 41.7 (37.8 to 46.0)	1.14 (0.98 to 1.32)	833	47.9 (42.3 to 54.2) vs 39.2 (35.0 to 43.9)	1.50 (1.25 to 1.81)

*Adjusted for sex, race, age at SLE diagnosis, education, geographic residence and the following variables assessed at time-zero: SLE duration, renal damage according to SLICC Damage Index, body mass index, smoking, prednisone, immunosuppressives and biologics. The main analysis was additionally adjusted by disease activity at time-zero.

†Remission was defined as SLEDAI-2K=0 and no prednisone or immunosuppressives use during the last year.

‡Low disease activity state was defined as SLEDAI-2K <4 and current prednisone dose ≤7.5 mg/day.

HCQ, hydroxychloroquine; SLE, systemic lupus erythematosus; SLEDAI-2K, SLE Disease Activity Index-2000; SLICC, Systemic Lupus International Collaborating Clinics.

Accounting for clustering and removing hospitalisation from the composite outcome led to small changes in the SEs, but had little or no effect on HR estimates (data not shown).

## Discussion

Ours is the first study in incident SLE to demonstrate that patients who reduced or discontinued HCQ had an increased risk of flaring versus those who maintained therapy. Other medications, geographic location and education were associated with flare risk. Age was not a clear risk factor, which is interesting given a recent paper that suggested HCQ discontinuation may be relatively safe in seniors (although the time-frame for flare risk was 1 year only).^8^ When stratifying our own results by age >50, power was decreased, but there remained a trend for HCQ maintenance being associated with a lower crude flare rate (25.3 events per 100 person-years, 95% CI 20.4 to 31.3) versus HCQ reduction (36.9 events per 100 person-years, 95% CI 30.0 to 45.4). The same trend was seen for HCQ discontinuation during person-time for age >50, again with imprecision (HCQ maintenance flare 31.0 events per 100 person-years, 95% CI 24.6,39.2 and HCQ discontinuation flare rate 42.4 events per 100 person-years, 95% CI (37.2 to 48.4).

Patients using immunosuppressives or prednisone at time-zero were at higher risk of flare after either HCQ maintenance, reduction or discontinuation. At least two other cohort studies have shown that patients under immunosuppressives (who generally have fairly severe SLE) have a twofold higher flare risk overall (vs patients not on immunosuppressives, who generally have less severe SLE).^21 22^ In addition to immunosuppressives, steroids are also markers of severe and active SLE.^23–25^

We observed some geographical differences in SLE treatment management and flare risks. Patients from Asia were more likely to reduce HCQ than maintain the dose (table 1). A survey showed that, compared with Europeans, Asian physicians were more likely to taper HCQ even in in patients with severe disease.^26^ Another study conducted in South Korea observed that polymorphisms in CYP2D6*10, an allele that is more common in Asians than in Caucasians,^27 28^ were associated with higher blood concentrations of HCQ’s metabolite.^29^ Together with recent results suggesting that Asian patients are more adherent to HCQ than Caucasians,^30^ this evidence may correlate with our finding that patients living in Asia had a lower risk of flaring after HCQ reduction than those living in North America and Europe. Since data from Asia came from a single tertiary centre in South Korea, these findings may reflect local practices or factors inherent to that population.

Low education was associated with increased flare risk among patients discontinuing HCQ. Low education is a well-known predictor of poor adherence to long-term therapies including in SLE.^31–34^ Subjects who discontinued HCQ (particularly those with low education) may have been non-adherent with other medications and physician advice, perhaps due to mistrust or not understanding physician recommendations.^31 35^

Our results suggest that HCQ maintenance typically results in lower flare risks, even in patients in disease remission. This finding is interesting in view of a small survey which suggested some (though not all) rheumatologists attempt to taper or discontinue HCQ in patients in remission^26^ and indicates that current disease activity alone may not sufficiently predict who will flare after HCQ is tapered. Incomplete adherence may explain some of our findings.^36^ However, flares occur even in patients with HCQ blood levels above the therapeutic threshold,^37^ reinforcing the relapsing-remitting nature of SLE, with durable remission being rare.^38 39^

The potential benefits of tapering or discontinuing HCQ must be balanced with the subsequent risk of a flare. Need for therapy augmentation occurred in 65%, 71% and ~40% of patients after HCQ discontinuation, reduction or maintenance, respectively. Of subjects needing therapy augmentation, over 65% augmented/started prednisone after HCQ reduction or discontinuation. Although the potential for antimalarial-induced toxicity (including retinopathy and cardiomyopathy) is of concern for patients and physicians,^4 40^ the adverse effects of glucocorticoids are severe and well established in patients with SLE^41^ and most physicians and patients would certainly prefer maintaining HCQ than augmenting prednisone.^42 43^

We studied a large international inception cohort with almost 20 years of follow-up and a well-characterised study population. However, some potential limitations should be mentioned. Patients and physicians did not explicitly provide the reason(s) for HCQ reduction or discontinuation. If decisions to reduce/discontinue HCQ are based on the patient’s current or past disease activity, long-term SLE remission may be more likely in the HCQ reduction/discontinuation cohorts, which may bias estimates towards the null. Despite this, our results still suggest that lowering/discontinuing HCQ is associated with higher flare risk versus maintaining HCQ.

Another potential limitation is that our composite outcome includes some interval-censored endpoints (those assessed only at annual clinic visits). However, simulations reported in the study by Huszti *et al*,^44^ for example, indicate that this will induce only minor bias towards the null in the estimated HRs. Moreover, our composite outcome is a practical approach similar to that used in clinical trials and, in addition to the accepted minimal clinically significant SLEDAI-2K change (important but not always sensitive), we included SLE-related hospitalisations (detecting the most serious SLE flares), as well as drug changes (a potentially more enduring marker of flares). Unfortunately, our definition of flare cannot clearly separate mild from moderate or severe flares.

It is interesting that the HCQ reduction and discontinuation cohorts had similar flares rates. Among those who reduced HCQ, the mean doses of those flaring versus not flaring were similar. This may reflect individual differences in drug metabolism or even in the amount of HCQ stored in body tissues. It has been suggested that doses under the maximum 400 mg/day (eg, 200 and 300 mg/day) still are potentially associated with less activity, thrombosis and survival.^6 45 46^ We did not evaluate HCQ levels (which are not part of usual care at most of our centres) or self-reported adherence. Nevertheless, in adjusting for sex, age, race/ethnicity, education and multiple medications, we accounted for factors that are themselves strong predictors of adherence.

The implications of our study findings are complex, with the decision to maintain or taper HCQ still being up to the patients and their physicians, through discussion of the trade-offs between the risk of disease flare, with the potential benefits of tapering HCQ. Our results should help facilitate this, by providing information about risks of flare associated with maintaining, reducing or stopping HCQ, and how and demographic factors (eg, disease activity, medications, education) may influence outcomes. These carefully quantified risks could be translated to improve patient education materials and discussions between healthcare providers and patients. Last but not least, the fact that there are over 30 flares per 100 person-years, even while remaining on HCQ, emphasises the ongoing need to optimise therapeutic options in SLE.

## Data Availability

All data relevant to the study are included in the article. n/a.
